# Anlotinib inhibits esophageal cancer malignancy by ameliorating the immune microenvironment

**DOI:** 10.1007/s12672-026-04457-8

**Published:** 2026-01-23

**Authors:** Zhao Minghong, Yuan Lin, Huang Xi, Xia Chunjun, Sun Xiaoyi, Meng Lingyun, Yang Junchang, Yan Wenyue

**Affiliations:** 1 Department of Oncology, Jianhu People’s Hospital, Yancheng, 224700 Jiangsu China; 2 Department of Oncology, Yancheng First Hospital, Affiliated Hospital of Nanjing University Medical School, The First People’s Hospital of Yancheng, No. 66 Renmin South Road, Yancheng, 224001 Jiangsu China

**Keywords:** Esophageal cancer, VEGFR2, Anlotinib, Immune infiltration

## Abstract

Esophageal cancer (EC) is a global health challenge with limited treatment and low efficacy. The role of vascular endothelial growth factor receptor 2 (VEGFR2) in EC has attracted significant attention. In this study, we found that anlotinib, an oral VEGFR2 inhibitor, exhibited a strong inhibitory effect on EC in vitro and in vivo. Moreover, VEGFR2 expression was positively correlated with infiltration levels of macrophages and dendritic cells (DCs) via comprehensive analysis and molecular experiment. Together, we evaluated the correlations between VEGFR2 expression and immune infiltration and explored the role of anlotinib on EC, providing a potential strategy for the intervention of EC.

**Supplementary Information:**

The online version contains supplementary material available at 10.1007/s12672-026-04457-8.

## Introduction

Esophageal cancer (EC) has gradually become a global health concern that poses a substantial threat to public health, especially in China [1]. Squamous cell carcinoma and adenocarcinoma, the major subtypes of EC, pose unique challenges in both diagnosis and treatment [2]. The limitations and inherent shortcomings of traditional therapies (surgical intervention and chemoradiotherapy) are becoming increasingly apparent. Emerging immunotherapies are offering a promising avenue for anticancer treatment. The combination of immunotherapy and chemotherapy is able to enhance the response rate and prolong the survival of patients [3]. However, the overall response rates of EC patients to immunotherapy remain low. Therefore, it is necessary to develop new and effective strategies to overcome this problem for improving the prognosis of EC patients.

Anlotinib is a multi-target tyrosine kinase inhibitor with potent anti-angiogenic properties [4]. It has been used in the treatment of various solid tumors by *targeting* vascular endothelial growth factor receptors (VEGFRs), fibroblast growth factor receptors (FGFRs), platelet-derived growth factor receptors (PDGFRs) and c-Kit. Many studies have indicated that anlotinib possesses the capacity to suppress tumor growth, angiogenesis and metastasis. Furthermore, previous studies demonstrated that anti-angiogenic agents have the capacity to modulate the tumour microenvironment [5–8]. Nevertheless, whether anlotinib has immunomodulatory effects in solid tumors remains unknown. Here, we reported that anlotinib exhibited proliferation-inhibiting effects in EC cells and inhibited tumor growth in vivo. The potential mechanism of anlotinib in EC was also explored, and it was shown that anlotinib may participate in immune infiltration, suggesting that anlotinib may enhance the efficacy of immunotherapy for EC patients.

## Materials and methods

### Cell culture and reagents

The human esophageal squamous cell carcinoma lines ECA109 (RRID: CVCL_6898) and KYSE150 (RRID: CVCL_1348) were obtained from the American Type Culture Collection (ATCC) in June 2023. Both cell lines were authenticated by short tandem repeat (STR) profiling and confirmed to be free of mycoplasma contamination by PCR-based testing prior to use in the study. These cell lines were cultured in DMEM (WISENT, Canada) medium supplemented with 10% fetal bovine serum (FBS, WISENT, Canada) and 1% penicillin/streptomycin (WISENT, Canada) at 37 °C in a 5% CO_2_ incubator. Anlotinib (AL3818) dihydrochloride was purchased from Selleck, and its stock solution was prepared according to the manufacturer’s instructions.

### IC50 assay

EC cells were cultured in 96-well plates and were dosed with the indicated anlotinib. IC50 values were calculated by fitting a dose–response curve to normalized data using GraphPad Prism software.

### Cell proliferation assay

Cell proliferation was measured using the CCK-8 kit according to the protocol recommended by the manufacturer (Dojindo Laboratories, Kumamoto, Japan).

### Quantitative real-time PCR

Total RNA were extracted from tumor tissues using the FastPure^®^ Cell/Tissue Total RNA Isolation Kit V2 (Vazyme, China) based on the manufacturer’s instructions and quantified with the NanoDrop spectrophotometer. HiScript^®^ Ⅱ Q RT SuperMix (Vazyme, China) and ChamQ SYBR qPCR Master Mix (Vazyme, China) were used for the production of complementary DNA and detection of targeted gene expression. For relative expression, each gene was normalized to β-actin using the ΔΔ*C*_t_ calculation and the 2^−ΔΔ*C*t^ method was used to calculate the fold change in gene expression between the tested samples. The primers used for real-time PCR are listed in Supplementary Table S1.

### In vivo mouse experiments

All animal studies were performed in compliance with the guidelines approved by the Animal Ethical and Welfare Committee of Jianhu People’s Hospital. Four-to-five-week-old BALB/c nude mice were obtained from Model Animal Research Center of Nanjing University. The KYSE150 cells (2 × 10^6^ cells) were subcutaneously injected into the flank region of the mice. When the average tumor volume reached approximately 100mm^3^, mice were divided randomly into two groups and given intragastric administration with saline or anlotinib at a dose of 3 mg/kg at 3-day intervals with a total of five times. During the experiment, the tumor volume was measured every three days using calipers, and the body weight of the mice was also recorded. The tumor volume was calculated using the following formula: tumor volume (mm3) = width^2^ ×length× 0.5.

### Prognosis analysis

The analysis between VEGFR2 expression and overall survival is based on published data analyzed by the KM-plotter (http://www.kmplot.com).

### Analysis of immune infiltration

The immunedeconv, an R package that integrates six of the latest algorithms for estimating immune cell concentrations, including CIBERSORT, EPIC, TIMER, MCPCOUNTER, QUANTISEQ, and XCELL was utilized to analyze the role of VEGFR2 in EC microenvironment infiltration based on TCGA database. Statistical analysis was conducted using R software, version v4.0.3. Results were considered statistically significant when the p-value was less than 0.05.

### Statistics

Experiments were repeated at least three times. Statistics analyses were performed using a student t test (two-tailed) or one-way ANOVA with GraphPad Prism seven. All results are presented as mean ± SD. (*p < 0.05; **p < 0.01; ***p < 0.001; ****p < 0.0001; ns, not significant)

## Results

### Anlotinib inhibited the proliferation of EC cells both in vitro and in vivo

**Fig. 1:**
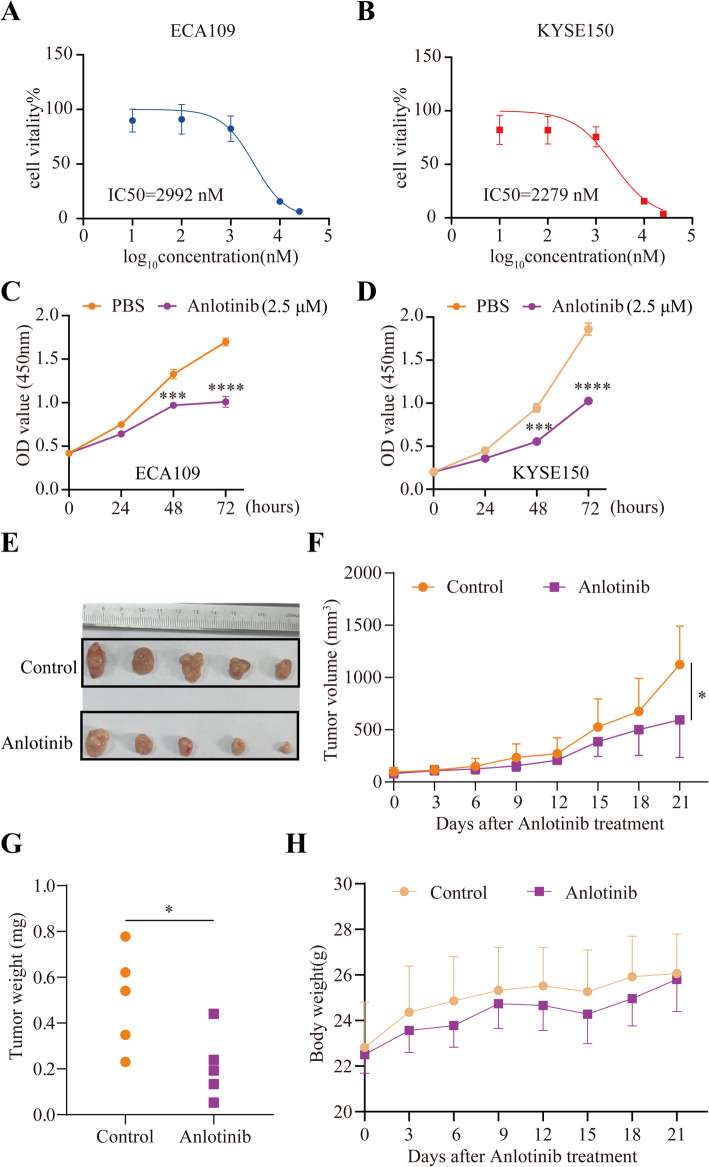
Anlotinib inhibited the proliferation of EC cells both in vitro andin vivo. **A**, **B** Anlotinib sensitivity assay showsthat the value of IC50 of ECA109 cells is 2992 nM and the value of IC50 of KYSE150 cells is 2279 nM. **C**, **D** Cell survivalassay shows that anlotinib can inhibit the growth of ECA109 cells and KYSE150 cells treated with 2.5μmol anlotinib.**E** Representative xenografted tumors from Anlotinib group and the control group. **F** The curves of tumor volumesof mice in the Anlotinib group and the control group as indicted time. **G** The tumor weight in the Anlotinib groupis significantly smaller than that in the control group. **H** The curves of body weights of mice in the Anlotinib groupand the control group as indicted time time. The t test was used for statistic quantifications: *p < 0.05, ***p < 0.001,****p < 0.0001

To investigate the role of anlotinib in EC, we first performed anlotinib sensitivity assays to obtain the IC50 values of EC cells, ECA109 cells (IC50 = 2992 nM) and KYSE150 cells (IC50 = 2279 nM) (Fig. 1A and B). Next, the effect of anlotinib on EC cell proliferation was evaluated using CCK-8 assay. We found that 2.5µM anlotinib exhibited significant inhibition of growth on EC cells (Fig. 1C and D). Then, we inoculated KYSE150 cells into the flank region of BALB/c nude mice to validate the inhibitory effect of anlotinib in vivo. The results showed that anlotinib treatment significantly shrank the KYSE150 tumors compared with PBS treatment (Fig. 1E –H). Together, our data demonstrate that anlotinib is able to inhibit EC progression both in vitro and in vivo.

### Anlotinib regulated immune infiltration through VEGFR2

**Fig. 2:**
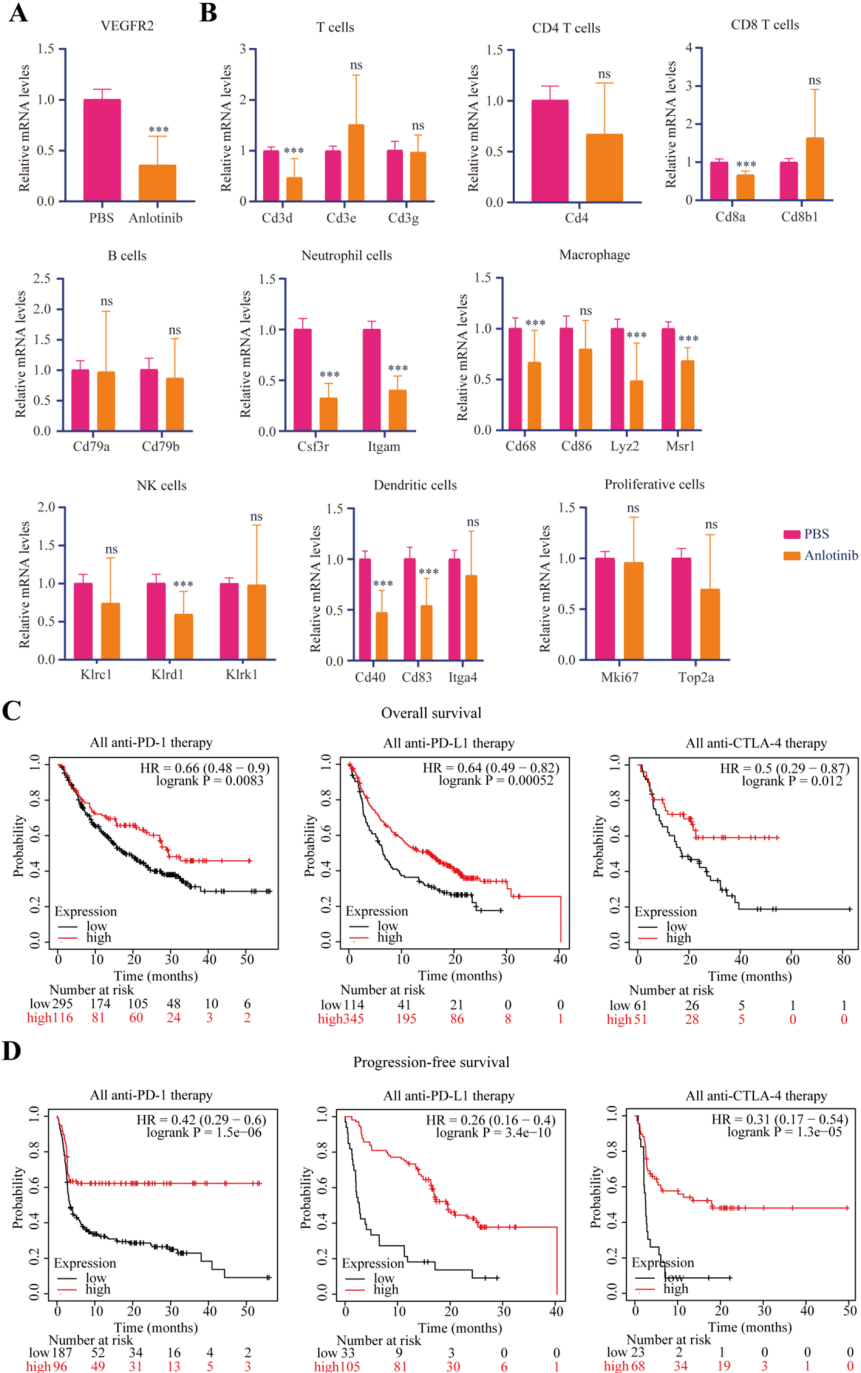
Anlotinib regulated immune infiltration through VEGFR2. **A** The mRNA level of VEGFR2 was examined by quantitative PCR. **B** The expression of marker genes related to immune cells was examined by quantitative PCR. **C** Analysis of public databases shows that the high expression of VEGFR2 is significantly associated with a high overall survival rate in anti-PD-1, PD-L1 and CTLA-4 immunotherapies. **D** Analysis of public databases shows that the high expression of VEGFR2 is significantly associated with a high disease-free survival rate in anti-PD-1, PD-L1 and CTLA-4 immunotherapies. The t test was used for statistic quantifications: ***p < 0.001, ns, not significant.

Previous studies have reported that VEGFR2 is the target site of anlotinib and VEGFR2 is associated with immune infiltration. To investigate whether anlotinib inhibits EC progression via modulating immune cell infiltration, we first ran the CIBERSORT, EPIC, TIMER, MCPCOUNTER, QUANTISEQ, and XCELL algorithm to acquire immune infiltration relationship with VEGFR2. We discovered that VEGFR2 presented strong positive correlated with macrophages, B cells, DCs and endothelial cells. And CD4 + memory T cells were negatively correlated with VEGFR2 in EC (Supplementary Fig. 1). Then, We examined the expression of marker genes related to immune cells in tumour tissues. We found that VEGFR2 expression was decreased in tumor tissues with anlotinib treatment (Fig. 2A). Moreover, the expression of marker genes related to macrophages and DCs were decreased in tumor tissues (Fig. 2B). These results suggest that anlotinib modulates immune cell infiltration. Kaplan-Meier survival analysis based on TCGA data demonstrated that high VEGFR2 expression was linked to better prognosis for cancer patients with immunotherapy (Fig. 2C and D). Together, our findings may help to elucidate the role of anlotinib in EC therapy, and can provide a strategy for improving immunotherapy in the future.

## Discussion

High surgical risks and low response in EC treatment led to an urgent need to develop new therapeutic regimens. The combination of immune checkpoint inhibitors and anti-angiogenic drugs has attracted significant attention for cancer treatment [9, 10]. Accumulating evidence suggest that combined drug regimen can bring remarkable survival benefits to patients with malignant tumors [11–16]. Anlotinib, as a representative anti-angiogenic drug, has shown that it can enhance anticancer effect and regulate PI3K/Akt signaling [17, 18]. Based on our results and previously reported on anlotinib and VEGFR2, the potential molecular mechanism for anlotinib to suppress cell proliferation maybe through the MAPK and AKT signaling pathways (Supplementary Fig. 2). Moreover, synergistic effect is observed in combination therapy with anlotinib and immunotherapy [19]. Therefore, a better understanding of anlotinib multifaceted effects may provide benefits for developing strategy for EC treatment.

In this study, we assessed the role of anlotinib in EC using in vitro and in vivo experiments combined with bioinformatics analysis, revealing that anlotinib is able to inhibit EC progression and regulates immune cell infiltration. Similar inhibitory effects of anlotinib were also found in lung cancer and gastric cancer [20–23]. Compared to previous studies in other cancers, our findings provide new effects of anlotinib on the immune microenvironment in EC [24–27]. Collectively, these findings strongly suggest that anlotinib could be part of therapeutic regimens.

The positive correlation between VEGFR2 expression and immunotherapy prognosis highlights the potential importance of VEGFR2 in immunotherapy. High VEGFR2 expression is associated with a favorable immune microenvironment that promotes the efficacy of immunotherapy. VEGFR2 is able to regulate the immune cell adhesion, the infiltration of immune cells, Treg proliferation and the cytotoxic activity of T cells [28]. Furthermore, VEGFR2 is reported to be involved in immune response via TNF-α signaling and PI3K pathway [28]. The relationship between VEGFR2 and different immune cell infiltrations revealed by various algorithms, further supports the idea that VEGFR2 is involved in the complex crosstalk between the tumor and the immune system. Understanding this relationship may provide new insights for developing more effective combination strategies of anlotinib and immunotherapy. Our study demonstrated the modulatory effect of anlotinib on the immune microenvironment, suggests that anlotinib may be a promising candidate for combination with immunotherapy in EC treatment. Future clinical trials could evaluate the safety and efficacy of such combinations. In addition, VEGFR2 may serve as a predictive biomarker for patients with immunotherapy. Therefore, anlotinib could be combined with immunotherapy for EC to facilitate a high efficacy treatment in the future.

## Conclusions

In summary, this study has investigated the effect of anlotinib on EC cells and the immune microenvironment. Anlotinib has shown significant inhibitory effects on EC cell lines in vitro and in vivo. It also modulates the immune microenvironment via affecting the infiltration of neutrophils, macrophages, and DCs. The positive correlation between VEGFR2 expression and immunotherapy prognosis, as well as between VEGFR2 and immune cell infiltration, suggests that anlotinib may enhance the efficacy of immunotherapy in EC. These findings provide a solid foundation for the development of novel treatment strategies for EC.

## Supplementary Information

Below is the link to the electronic supplementary material.

Supplementary Material 1.

Supplementary Material 2. Supplementary Figure 1. Analysis of the Correlation between VEGFR2 Expression and Immune Infiltration. (A-F) CIBERSORT, EPIC, TIMER, MCPCOUNTER, QUANTISEQ and XCELL algorithm calculation of immunocyte infiltration in EC. Supplementary Figure 2. A diagram to illustrate the potential molecular mechanism for the inhibitory effect of anlotinib on EC cell lines.

## Data Availability

All data and materials supporting the findings of this study are available from the corresponding author upon reasonable request.
