# Supplementary material for: Anlotinib inhibits esophageal cancer malignancy by ameliorating the immune microenvironment
Source: Discov Oncol. 2026 Jan 23;17:320. doi: 10.1007/s12672-026-04457-8 (PMC12913843; doi:10.1007/s12672-026-04457-8)
Supplement: Supplementary file 2 — Supplementary Material 2. Supplementary Figure 1. Analysis of the Correlation between VEGFR2 Expression and Immune Infiltration. (A-F) CIBERSORT, EPIC, TIMER, MCPCOUNTER, QUANTISEQ and XCELL algorithm calculation of immunocyte infiltration in EC. Supplementary Figure 2. A diagram to illustrate the potential molecular mechanism for the inhibitory effect of anlotinib on EC cell lines. [file 12672_2026_4457_MOESM2_ESM.docx]

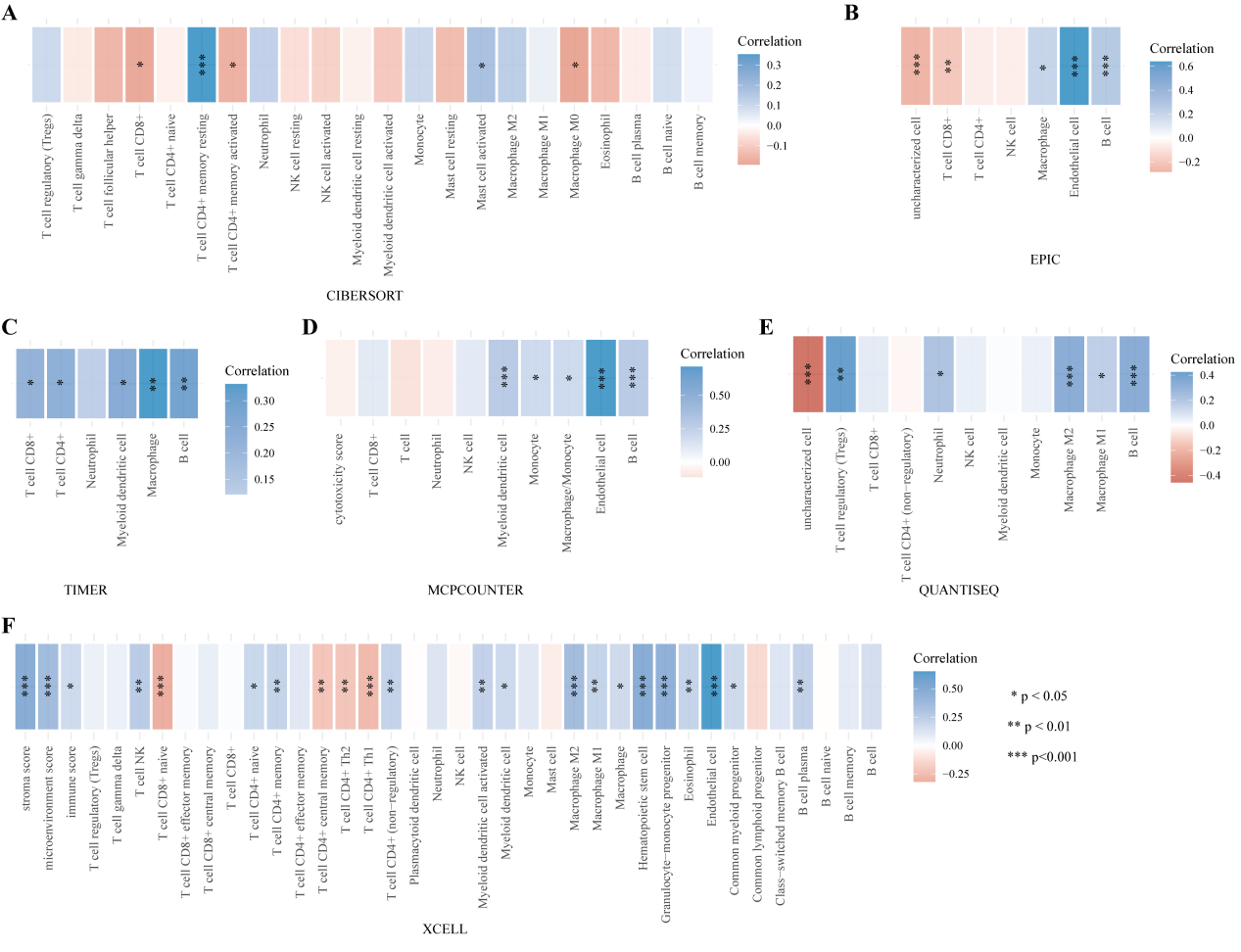


**Supplementary Figure 1. Analysis of the Correlation between VEGFR2 Expression and Immune Infiltration.** (A-F) CIBERSORT, EPIC, TIMER, MCPCOUNTER, QUANTISEQ and XCELL algorithm calculation of immunocyte infiltration in EC.


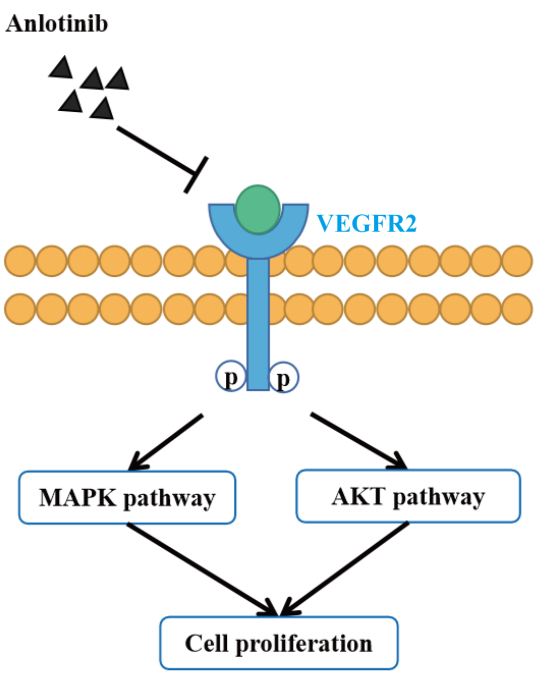


**Supplementary Figure 2.** A diagram to illustrate the potential molecular mechanism for the inhibitory effect of anlotinib on EC cell lines.
